# A silver bullet for ageing medicine?: clinical relevance of T-cell checkpoint receptors in normal human ageing

**DOI:** 10.3389/fimmu.2024.1360141

**Published:** 2024-02-01

**Authors:** Lucy Rimmer, Derek A. Mann, Avan A. Sayer, Shoba Amarnath, Antoneta Granic

**Affiliations:** ^1^ AGE Research Group, Translational and Clinical Research Institute, Faculty of Medical Sciences, Newcastle University, Newcastle upon Tyne, United Kingdom; ^2^ Newcastle Fibrosis Research Group, Biosciences Institute, Newcastle University, Newcastle upon Tyne, United Kingdom; ^3^ National Institute for Health and Care Research (NIHR) Newcastle Biomedical Research Centre, Newcastle upon Tyne Hospitals National Health Service (NHS) Foundation Trust and Newcastle University, Newcastle upon Tyne, United Kingdom; ^4^ Newcastle University Biosciences Institute, Medical School, Newcastle University, Newcastle upon Tyne, United Kingdom

**Keywords:** T-cell, checkpoint, healthy ageing, immunosenescence, translational research

## Abstract

Immunosenescence describes dysregulation of the immune system with ageing manifested in both the innate and adaptive immunity, including changes in T-cell checkpoint signaling. Through complex and nuanced process, T-cells lose excitatory signaling pathways and upregulate their inhibitory signaling, leading to ineffective immune responses that contribute to the formation of the ageing phenotype. Here we expand on the expression, function, and clinical potential of targeting the T-cell checkpoint signaling in age and highlight interventions offering the most benefits to older adults’ health. Notably, modifications in vaccination such as with mTOR inhibitors show immediate clinical relevance and good tolerability. Other proposed treatments, including therapies with monoclonal antibodies fail to show clinical efficacy or tolerability needed for implementation at present. Although T-cell co-signaling fits a valuable niche for translational scientists to manage immunosenescence, future study would benefit from the inclusion of older adults with multiple long-term conditions and polypharmacy, ensuring better applicability to actual patients seen in clinical settings.

## 1 Introduction

A “silver bullet” targeting multiple facets of the ageing phenotype would revolutionize the field of ageing medicine. Immune system dysregulation with age, known also as immunosenescence, is one such target for universally treating the consequences of ageing (1). Immunosenescence has been linked to worsened infection control, insufficient vaccination response, reduced tumor surveillance, and a paradoxical increase in rates of autoimmunity in older adults (2, 3). Links between immunosenescence and other aspects of the ageing phenotype continue to be explored (4). Questions remain whether targeted reversal of immune ageing at a cellular level can produce clinically relevant effects.

This review aims to provide physician scientists with an overview of translationally relevant research relating to T-cell co-receptor signaling in age, including discussions around applicability and feasibility of proposed treatments. A summary of normal immune cell interactions will be described, as well as a brief overview of general age-related changes in the immune system. More detailed illustration of functions, changes with age, and clinical relevance of four key co-receptors (CD28, ICOS, CTLA-4 and PD-1) will then be described, as opposed to a comprehensive summary of all co-receptor changes with age.

## 2 Overview of age-related changes in the immune system

Although describing all known aberrations in the ageing immune system is unfortunately beyond the scope of this review, some contextualization remains valuable. Breaking down of normal intercellular signaling in the innate immune system occurs in a complicated web of immune alterations including, non-exhaustively, shifting T-lymphocyte subsets, B-cell dysfunction and dysregulated cytokine production (3). T-cell co-receptor expression patterns and signaling therefore serve as one of many interlinked changes in an ageing immune system and should be considered as such.

**Figure 1:**
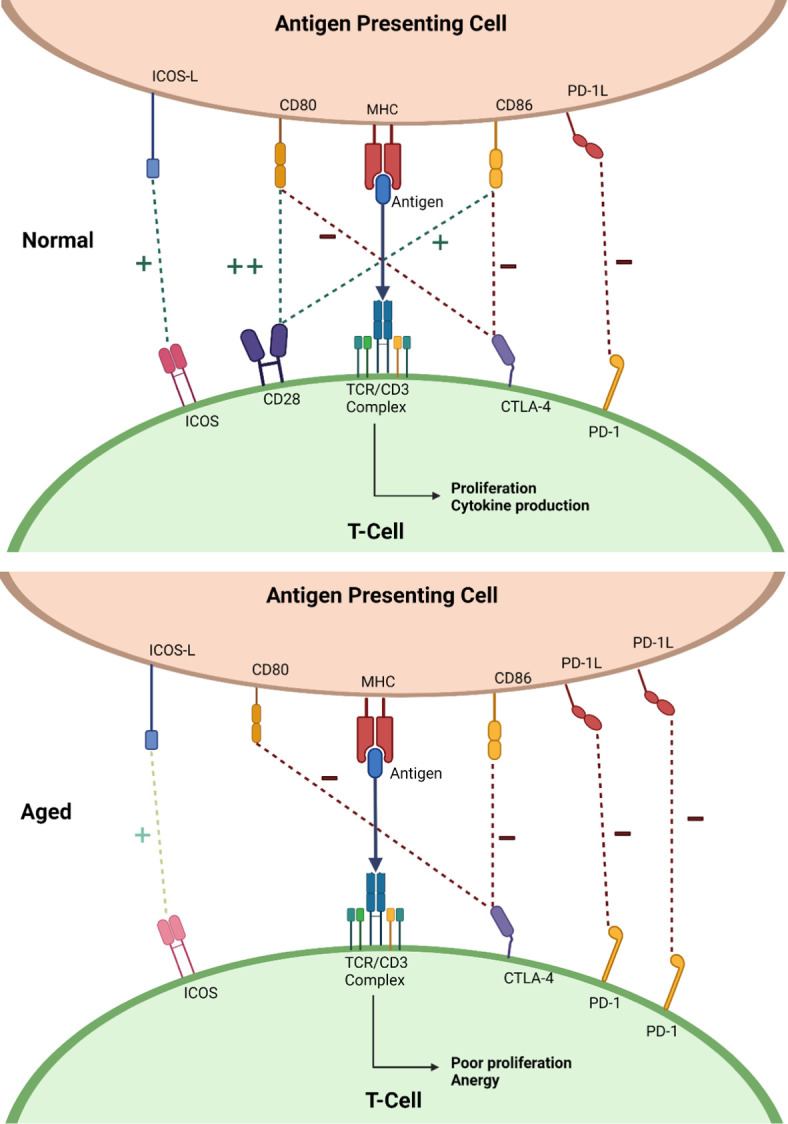
Diagrams showing a simplified representation of normal co-signaling seen in the young (top) and the changes seen in ageing (bottom). “+” with the dark green dotted line represents positive co-signaling and “-” with the dark red dotted line represents negative co-signaling. Abbreviations: inducible T-cell co-stimulator (ICOS), cytotoxic T-lymphocyte associated protein 4 (CTLA-4), programmed death receptor-1 (PD-1), cluster of differentiation 28 (CD28), T-cell receptor (TCR), cluster differentiation 3 (CD3), major histocompatibility molecule (MHC), cluster differentiation 80 (CD80), cluster differentiation 86 (CD86), inducible T-cell costimulatory ligand (ICOS-L), programmed death receptor ligand-1 (PD-1L). Created with BioRender.com.

Changes occur in both the innate and adaptive immune system with age, though it is aberrations in the latter that will make up the focus of this review. B-lymphocytes and T-lymphocytes are the key cells in the adaptive immune system. Under normal circumstances, a “first signal” is produced by an antigen presenting cells (APC) presenting a novel antigen on its major histocompatibility complex (MHC) to the T-cell receptor (TCR) on the T-lymphocyte. Response to the antigen is dependent on the “second” signal or “co-signal”, arising from receptor-ligand interactions between the T-lymphocyte and the APC. Second signals can either encourage response to the novel antigen, co-stimulation, or encourage tolerance and prevent a response, co-inhibition. Balancing these two opposing signals allows for a strong immune response to threatening antigens, whilst allowing tolerance to self-antigens. **Figure 1** demonstrates in more detail how this sophisticated process devolves with age.

## 3 Changes in ageing

### 3.1 Co-stimulatory molecules

#### 3.1.1 CD28

CD28 is part of the wider B7-superfamily of receptors, serving as a primary co-stimulatory signaling molecule on T-cells. When activated by its complementary ligands CD80 and CD86 on the APC, CD28 enhances proliferation and promotes the production pro-inflammatory cytokines in response to an antigen. Moreover, CD28 employs autocrine signaling via IL-2 to sustain and enhance its cell surface expression upon activation, thereby prolonging its functional capacity (5).

CD28 is almost ubiquitously expressed on T-lymphocytes in younger subjects, however a noticeable pattern of reduced expression emerges with age (6, 7). This decline in CD28 cell surface expression begins early in the ageing process, leading to a reversible CD28null state, before progression to an irreversible loss, forming a permanent CD28null state. Mechanistically, CD28 loss is attributed to a direct translational blockade at the promoter region of the CD28 gene, though deciphering the exact mechanistic cause of this age-related translational blockade is challenging (8). CD28null cells exhibit an “exhausted” phenotype, characterized by terminally differentiated, anergic T-cells with shortened telomeres (9–11). Cellular exhaustion occurs naturally as cells approach their Hayflick limit (the maximum number of times a cell can divide before reaching replicative senescence) during the cellular ageing process. The process of exhaustion can be accelerated when T-cells are required to be active over a prolonged period of time, notably seen in T-cells involved in preventing the reactivation of chronic viral infections such as Cytomegalovirus (CMV) and varicella zoster (VZV) (12–16). The immune system uses significant resources throughout life to prevent reactivation, leading to an increased rate of immune cell proliferation and subsequent exhaustion with loss of excitatory checkpoint receptors (12–16).

Another potential contributing factor to CD28 decline is increased exposure to inflammatory cytokines with ageing, relating to the inflammageing phenomenon. These cytokines can directly trigger a transcriptional blockade; TNF-α in particular appears the most probable causative cytokine in this process (17–20).

Additionally, cell surface expression alone fails to capture the full scope of changes in CD28 function with age. Even in cells where CD28 expression is maintained, functional deficits can arise from an inability to relocate CD28 to the immune synapse, the crucial site of antigen presentation (21). Ineffective cholesterol efflux mechanisms in aged T-lymphocytes cause stiffening of the phospholipid bilayer, hindering the migration of CD28 to the immune synapse upon activation (22). Consequently, there is a functional loss of CD28 co-stimulation despite retention of cell surface expression. (22).

##### 3.1.1.1 Modifying CD28 expression in ageing

Preventing CD28 loss could be the key strategy to combat immunosenescence. Simple interventions may include vitamin E supplementation, which could potentially increase IL-2 production and thus cause CD28 upregulation (23). Another approach to explore would be the dietary use of high-density lipoproteins (HDL) which can improve membrane fluidity in T-cells. Increased fluidity would allow for improved CD28 migration to the immune synapse (22). While the available evidence for HDL treatment in older T-cells *in vitro* has been somewhat underwhelming when compared to younger T-cells, it still showed modest functional gains in both groups (22).

High intensity interval training (HIIT), which involves short bursts of intense exercise followed by periods of rest, has shown promise as an intervention. HIIT may selectively mobilize CD28null cells from peripheral tissues and encouraging their apoptosis, and hence clearing them from circulation and allowing for the expansion of more functional CD28+ cells. Importantly, HIIT offers a non-pharmacological or dietary-based intervention for immune ageing (24).

Medical interventions aimed at targeting chronic viral exposure are crucial towards the prevention or improved management of infections in the later stages of life. Vaccination for VZV is available and clinically effective, however CMV, which is suspected to be the larger contributor of T-cell exhaustion and CD28 loss, remains without an approved vaccination. Promising CMV vaccines are currently under investigation in trials, however (25). Where prevention is not possible, management of chronic viral infections continues to be a clinical challenge. Letermovir, a CMV antiviral medication commonly used in a transplant setting, has good tolerability and minimal few side effects (26). While the use of antiviral medications for preventing immunosenescence has yet to be formally explored, it presents an exciting avenue worthy of investigation.

Interventions aimed at modifying cytokine pathways hold potential, though are currently at an experimental stage. As an example, upregulating IL-21 could promote direct upregulation of CD28 at a transcriptional level (27). While drugs targeting the IL-21 pathway are coming into use in the management of certain malignancies, there are no studies directly utilizing them to manage immunosenescence (28).

Radical removal of CD28null cells allows offers an interesting approach, as it would theoretically allow for creation of an “immunological space” for naïve CD28+ cells to expand. Notably, CD28null cells do not contain unique clonotypes, so *in vitro* studies have suggested that their removal should not impact immunological memory (16). However, no methods of CD28null removal *in vivo* currently exist, so this work remains largely hypothetical.

#### 3.1.2 Inducible co-stimulator

Inducible co-stimulator (ICOS), another member of the B7-superfamily, serves as the second most important co-stimulation molecule. Upon T-cell activation, ICOS expression is upregulated and provides a positive co-signal by interacting with its ligand ICOS-L (B7-H2) on APCs. Evidence surrounding ICOS expression with age is mixed. While reduced ICOS expression in aged T-cells compared to young T-cells has been demonstrated, other studies show ICOS potentially compensates for CD28 loss in progressive population doublings, becoming the predominant co-signaling molecule in this context (29–35).

##### 3.1.2.1 Modifying ICOS expression in ageing

ICOS remains relatively under-explored from a translation perspective. However, a promising *in vivo* study in older adults exposed to higher-dosage influenza vaccination showed enhanced clinical protection, likely via increased ICOS expression in CD4+ T-cells (36).

A further intriguing concept is targeting of the dual specific phosphatase 4 (DUSP4). DUSP4 is a stress and activation-induced phosphatase which acts to reduce expression of ICOS. Vaccination counterintuitively reduces ICOS expression in older adults, which appears to be related to an upregulation of DUSP4 (33). DUSPs are a potential drug target emerging in the oncology field and hence may open-up new ways to modify vaccination in older adults in the future (37). However, a degree of caution may be required as global phosphatase inhibitors that are non-specific to co-receptors may cause changes, both positive and negative, to the ageing physiology.

### 3.2 Co-inhibitory molecules

#### 3.2.1 Cytotoxic T-lymphocyte associated protein 4

Cytotoxic T-lymphocyte associated protein 4 (CTLA-4) is an important co-inhibitory signal in T-cells. As part of the immunoglobulin superfamily, CTLA-4 competes with CD28 for its co-ligands CD80/CD86 on APCs. Upon TCR activation, increased CTLA-4 expression is stimulated, serving to dampen the immune response. In general, CTLA-4 expression increases with age. CD28 loss with age correlates with a gain in CTLA-4, compounding the negative effects on T-cell signaling (5, 20, 32, 34, 38–40). However, some studies disagree with this conclusion, indicating a loss of CTLA-4 with age, although this effect was limited to the CD8+ T-cell subset (30, 41).

##### 3.2.1.1 Cytotoxic T-lymphocyte associated protein 4

At the present time, evidence is sparse for interventions targeting CTLA-4 in the context of immunosenescence. Horticultural therapy, the use of gardening for health and rehabilitation, decreases CTLA-4 expression in older adults, possibly via reduction of circulating IL-6 (42). However, this conclusion has emerged only from a small pilot study (42). The use of CTLA-4 monoclonal antibodies allows for a more direct blockade of CTLA-4. Unfortunately, studies in older adults in cancer trials reflect mixed results from the clinical use of this approach. Various factors, including gut microbiome, are thought to affect efficacy of the treatment, which complicates application to wider patient groups (43). Further investigation at a pre-clinical and clinical investigations level is required to explore CTLA-4’s potential as a target for managing age-related immune dysfunction.

#### 3.2.2 Programmed cell death protein 1

Programmed cell death protein-1 (PD-1) plays dual roles in T-cell function, serving as both a co-inhibitory receptor and a coordinator of apoptosis. Upregulation of PD-1 occurs with TCR activation, providing a negative co-signal upon cross-linking with PD-1 ligand (PD-1L) or PDL-2, both of which are members of the extended CD28-superfamily.

PD-1 expression tends to increase with age. Similar to CD28, there is ongoing debate surrounding whether increased PD-1 expression arises from an “exhausted” phenotype or if age itself independently drives the upward trend in expression (44). Some evidence suggests that PD-1 cell surface expression does increase with age, even when accounting for factors such as CMV seropositivity (5, 32, 45). Irrespective of the exact cause, increased PD-1 expression significantly contributes to immunosenescence, as demonstrated elegantly by *in vitro* experiments that showed significantly improved proliferative response and antigen binding in aged T-cells exposed to PD-1-blockade (46–48).

##### 3.2.2.1 Programmed cell death protein 1

PD-1 has garnered substantial translational interest, this a reflection of the successes of immune checkpoint therapies across various clinical fields, most prominently oncology. Preventing PD-1 overexpression through interventions in earlier life offers a conservative management option for promoting a robust immune system in later life. Avoiding adiposity and a sedentary lifestyle in middle age offsets immunosenescence in older age, notably by reducing PD-1 (24). offsetting the adverse effects of chronic viral infections such as CMV and VZV may help avoid the exhausted T-cell phenotype that is functionally associated with immune decline with ageing, similar to CD28 (48).

Pharmaceutical options for modifying PD-1 exist through mTOR inhibitors, a very prominent drug class in the senescence and ageing fields. A randomized controlled trial of an mTOR inhibitor (RAD001, also known as Everolimus) in influenza vaccine response in older adults showed significantly improved antibody titres in participants randomized to treatment group (49). This correlated to lower PD-1 expression post-vaccination in the treatment group compared to baseline. Moreover, adverse events in the treatment group were limited and comparable to the control group during the 6-week trial period, indicating a favorable safety profile. More invasive pharmaceutical options exist through PD-1 monoclonal antibodies, such as Nivolumab (43). While these antibodies are more targeted than mTOR inhibitors, they exhibit worse efficacy and more significant adverse events in older adults than younger counterparts (50). Postulated reasons for lack of efficacy of Nivolumab in older adults includes lack of concurrent CD28 expression, which evidence indicates needs to be maintained for full benefit of the treatment.

## 4 Discussion

Co- receptor signaling in normal human ageing presents with reduced expression and function of co-excitatory signals from CD28 and ICOS, and a concurrent increase in co-inhibitory signaling via CTLA-4 and PD-1. Treatments that recover co-receptor signaling in age to alleviate the ageing phenotype remains largely theoretical, with only some relating to vaccination offering immediate clinical relevance.

Limited options exist that could immediately integrate into clinical practice. Simple interventions in vaccination that improve clinical protection, such as using higher dosage vaccinations, would be easily implementable at a large scale (51). Use of mTOR inhibitors in vaccination also has some of the best evidence of clinical efficacy, tolerability and potential for imminent use (49). Although the intervention was well tolerated, with few adverse effects compared to control, older adults in a standard clinical setting experience polypharmacy, putting them at higher risk of side effects compared to the study population. The aforementioned interventions, though encouraging, fail to fill the role as a “silver bullet” succeeding only in improving vaccination response.

Other interventions, including horticultural therapy, HIIT and maintaining a non-sedentary lifestyle, benefit from their non-pharmacological nature and ease of application. However, these interventions show only modest biochemical improvements in co-signaling and lack evidence of clinical outcomes. Future studies would benefit from combining biochemical outcomes with clinical outcomes, for example vaccination response or rates of infections.

Novel treatments are travelling down the pipeline. Monoclonal antibody therapies possess significant drawbacks, including adverse events and reduced efficacy in old adults (43, 50). However, they are highly targeted treatments and with optimization of dosages and treatment regimens, they may prove to be the aforementioned “silver bullet” for co-signaling in older adults. But much work needs to be completed before this is the case.

Transferring findings of this review to clinical practice presents limitations. Focusing only on healthy older adults limits its applicability to a more typical older adult seen in a healthcare setting, who increasingly suffer from multiple long-term conditions and polypharmacy. It may indeed be the case that the evidence underestimates both the changes that occur in co-signaling with age and the effectiveness of interventions, as patients included are more immunologically competent due to their good health. Older adults may struggle to practically engage in treatments including exercise regimens or horticultural therapy due to underlying conditions, or to regularly attend hospital appointments for monoclonal antibody infusions. Therefore, the interventions offered in this review need contextualizing to the patient cohort and the risk-benefit of treating seriously considered.

Normal human ageing presents complex changes to T-cell co-signaling and in the immune system as a whole and certainly further characterizing of these changes is needed. Clinical relevance of T-cell co-signaling changes are still being revealed.

## 5 Conclusion

Changes occurring within co-receptor signaling in normal human ageing continues to be an under-researched area. Clinical relevance of co-signaling changes also requires require further exploration. Some treatments, notably mTOR inhibitors in vaccination, show more immediate clinical relevance. Finally, all further studies would benefit from the inclusion of older adults with multiple long-term conditions and polypharmacy, ensuring better applicability to how patients in this age group present in clinical settings.

## Author contributions

LR: Conceptualization, Data curation, Formal analysis, Investigation, Methodology, Writing – original draft, Writing – review & editing. DM: Supervision, Writing – review & editing. AS: Conceptualization, Supervision, Writing – review & editing. SA: Conceptualization, Supervision, Writing – review & editing. AG: Conceptualization, Supervision, Writing – review & editing.
